# Beyond the child: Integrating caregiver mental health into paediatric HIV and tuberculosis care in Africa

**DOI:** 10.1371/journal.pmed.1005287

**Published:** 2026-11-12

**Authors:** Adwoa Kumiwa Asare Afrane, Marilyn Naana Ahun

**Affiliations:** 1 Department of Child Health, University of Ghana Medical School, Accra, Ghana; 2 Department of Child Health, Korle Bu Teaching Hospital, Accra, Ghana; 3 Department of Medicine, McGill University, Montréal, Canada; 4 Child Health and Human Development Program, Research Institute of the McGill University Health Centre, Montréal, Canada

## Abstract

In this Perspective, Adwoa Afrane and Marilyn Ahun outline why greater attention is needed to the mental health of caregivers for children with HIV and/or tuberculosis, and discuss how recent policy commitments in Africa, including the Accra declaration, offer a rare opportunity for meaningful action.

## Teaser

Paediatric HIV and tuberculosis care in Africa pays little attention to children’s caregivers, although their mental health matters for child outcomes and family wellbeing. We argue that new continental and Ghanaian policy commitments offer a rare chance to make the family the unit of care.

More than seven decades after the World Health Organization (WHO) defined health as a state of complete physical, mental and social wellbeing, health systems across Africa still tend to focus on physical health, fragmenting care into vertical, disease-specific programmes. Paediatric HIV and tuberculosis (TB) make the consequences of this especially visible: both are major causes of chronic illness among children in Africa, both require months to years of daily treatment that an adult must supervise, and both carry stigma that reaches the whole household. Yet, while paediatric HIV and TB clinics are understandably built to optimise outcomes in the infected child (e.g., virological suppression), little systematic attention is paid to the wellbeing of the adults who raise them.

What gets lost in that singular focus is the person sitting beside the child. The caregiver (parent, grandparent or other relative) is not merely a proxy for the child. The health and wellbeing of the people around the child matter for child outcomes, as well as being an important goal in itself [1]. Many caregivers attending paediatric HIV clinics are themselves living with HIV, and many also experience bereavement, stigma, poverty and food insecurity, which are well-recognised social determinants of common mental disorders. Results from the SANKOFA paediatric HIV disclosure trial in Ghana have revealed how closely caregivers’ wellbeing and children’s care are intertwined: depressive symptoms were common among caregivers [2], children’s depression scores were correlated with those of their caregivers [3] and caregiver depressive symptoms and other psychosocial factors changed alongside the disclosure process [4]. Yet outside the research protocol, caregivers’ mental health was not routinely assessed or supported as part of paediatric HIV care. The clinical, social and economic costs of that gap fall first on caregivers themselves, but also on their children.

## Caregiver mental health matters for caregivers and for children

Mental health conditions, including depression, are recognised barriers to antiretroviral therapy adherence among adults living with HIV [5], and many caregivers of children with HIV are managing their own treatment as well as their child’s. The evidence linking caregiver mental health to children’s own treatment outcomes is less consistent: in one South African cohort, caregiver alcohol use and whether the primary caregiver was the child’s biological mother predicted children’s adherence more strongly than caregiver depression did [6]. In paediatric TB, research on caregiver mental health is sparse, even though caregivers supervise months of daily treatment. Taken together, these findings point less to any single diagnosis than to the combined psychological, social and material strain on the adults who manage children’s daily treatment, and to undetected or untreated caregiver depression as one part of that strain.

Beyond adherence, caregiver mental health shapes the caregiving environment more broadly. Maternal depression is associated with disrupted attachment, poorer feeding practices and developmental delays in young children, compounding the vulnerabilities already created by HIV or TB exposure [1]. Research in Ghana points to similar pathways. In a study of over 4,700 children and caregivers in Northern Ghana, caregiver psychological distress was negatively associated with children’s academic achievement and socioemotional outcomes; among families where caregivers had no formal education, reduced caregiver engagement in education partly explained the association with children’s literacy [7].

The SANKOFA trial also shows what careful attention to caregivers can make possible. Its disclosure intervention, which supported caregivers to prepare for and carry out disclosure of the child’s HIV status, improved children’s immunological outcomes at 48 weeks [8] and did not worsen depression scores in children or caregivers [3]. SANKOFA was not designed to test a caregiver mental health intervention, however, and whether identifying and treating caregiver depression improves children’s virological or TB treatment outcomes remains an open question. Routine depression screening on its own also has an inconsistent evidence base, particularly where it is not linked to treatment and follow-up [9]. The case for action therefore does not rest on screening; it rests on caregivers’ own need for care, and on established models for integrating mental health into HIV services (see Box 1) [5].

**Box 1. The Accra Declaration: A continental mandate.**

In July 2026, Ghana hosted the 20th Extraordinary Session of the African Union (AU) Assembly, dedicated to health. The resulting Accra Declaration commits member states to fully integrating HIV, TB, mental health conditions and other priority conditions within primary healthcare and universal health coverage service packages, moving away from parallel vertical programmes toward a unified approach [10]. For children living with HIV or TB across the continent, and the families who care for them, this commitment could be transformative if it is followed through.

For Ghana, the Declaration carries particular weight. The revised Mental Health Bill is pending parliamentary approval as of October 2026, and the country’s sparse mental health specialist workforce remains concentrated in Accra, out of reach for most families attending district-level paediatric HIV or TB clinics. Ghana is not starting from scratch, however. As a participating country in the WHO Special Initiative for Mental Health since 2021, it has worked with WHO and partners to build capacity at primary health facilities for early detection, treatment and psychosocial support, and since November 2024 the National Health Insurance Scheme has covered depression, anxiety disorders, bipolar disorder and schizophrenia [11]. What the Declaration adds is accountability: treating integration as a political commitment rather than an aspiration, something domestic advocacy alone has struggled to achieve. This window, with both continental and domestic policy frameworks aligned, is rare and narrow. Implementation must begin now.

## Ghana’s policy convergence

The Accra Declaration (see Box 1) arrives alongside Ghana’s first Maternal Mental Health Policy (2026–2037), launched in April 2026. The policy embeds mental health screening and support into routine antenatal and postnatal care, equipping community health nurses and midwives at Community-based Health Planning and Services compounds to recognise and respond to emotional distress [12]. We argue that its logic should extend to paediatric HIV and TB clinics too, where caregivers face sustained psychological and social pressures of their own beyond the perinatal period, and where their wellbeing is closely tied to their child’s care [2–4]. The same primary care platforms, strengthened through the WHO Special Initiative, provide shared infrastructure that could make this integration feasible.

## Learning from the continent

Other countries on the continent have already tested pieces of this model. In Uganda, group support psychotherapy delivered by trained lay health workers reduced depression among adults living with HIV in a cluster-randomised trial and was cost-effective [13]. In Zimbabwe, the Friendship Bench, a problem-solving therapy delivered by lay health workers in primary care clinics where many attendees were living with HIV, reduced symptoms of common mental disorders in a randomised trial [14]. What these programmes had in common was not specialist resources, but task-sharing, structured psychological support, supervision, and clear referral pathways. Most, however, were designed for adults attending their own care, and few have been adapted for caregivers who bring children to HIV or TB clinics, a gap that implementation research in Ghana could help to fill.

## Recommendations

Turning the Accra Declaration into more than a statement of intent will take concrete steps within Ghana’s paediatric HIV and TB programmes, built on existing models rather than new, parallel structures. First, national paediatric HIV and TB guidelines should state that caregiver mental health needs to be considered alongside the health of the child, adopting a family-centred approach in line with WHO and Joint United Nations Programme on HIV/AIDS (UNAIDS) guidance on integrating mental health and HIV services [5]. Where brief tools are used to identify caregivers in distress, they should be introduced alongside a clear care pathway, trained staff and follow-up, with their acceptability, feasibility and cost evaluated [9]. Second, the National AIDS/STI Control Programme and the National Tuberculosis Control Programme should work with the Mental Health Authority and the Ghana Health Service to extend the primary care training under way through the Special Initiative [11], based on WHO mhGAP Intervention Guide, to nurses and lay counsellors in paediatric HIV and TB clinics. Doing so would enable caregivers to receive psychosocial support and referral when they need it. Third, because caregiver distress is rooted in social, as well as clinical conditions, clinics should link families to social protection and support, including National Health Insurance Scheme enrolment, the Livelihood Empowerment Against Poverty cash transfer programme, and peer support groups that address stigma. Fourth, the principles of the Maternal Mental Health Policy should be extended to paediatric HIV and TB settings and to caregivers beyond the perinatal period, so that caregiver mental health sits within one policy framework rather than several separate ones. Finally, these steps should be evaluated for their effects on caregivers’ own wellbeing as well as on children’s outcomes.

## Conclusions

A child cannot thrive if the caregiver beside them is struggling in silence, and that caregiver deserves care in their own right. Ghana’s paediatric HIV and TB programmes have built strong infrastructure over the past two decades. Making room for caregivers’ mental health within existing consultations, backed by the primary care mental health services Ghana is already building, is not a new programme. It is an acknowledgement that the family, not the child alone, is the unit of care.
